# Reed Warbler Hosts Fine-Tune their Defenses to Track Three Decades of Cuckoo Decline

**DOI:** 10.1111/evo.12213

**Published:** 2013-08-08

**Authors:** Rose Thorogood, Nicholas B Davies

**Affiliations:** 1 Department of Zoology, University of CambridgeCambridge, United Kingdom

**Keywords:** Brood parasitism, coevolution, environmental change, phenotypic plasticity

## Abstract

Interactions between avian hosts and brood parasites can provide a model for how animals adapt to a changing world. Reed warbler (*Acrocephalus scirpaceus*) hosts employ costly defenses to combat parasitism by common cuckoos (*Cuculus canorus*). During the past three decades cuckoos have declined markedly across England, reducing parasitism at our study site (Wicken Fen) from 24% of reed warbler nests in 1985 to 1% in 2012. Here we show with experiments that host mobbing and egg rejection defenses have tracked this decline in local parasitism risk: the proportion of reed warbler pairs mobbing adult cuckoos (assessed by responses to cuckoo mounts and models) has declined from 90% to 38%, and the proportion rejecting nonmimetic cuckoo eggs (assessed by responses to model eggs) has declined from 61% to 11%. This is despite no change in response to other nest enemies or mimetic model eggs. Individual variation in both defenses is predicted by parasitism risk during the host’s egg-laying period. Furthermore, the response of our study population to temporal variation in parasitism risk can also explain spatial variation in egg rejection behavior in other populations across Europe. We suggest that spatial and temporal variation in parasitism risk has led to the evolution of plasticity in reed warbler defenses.

Although alternative genotypes can change in frequency over time as selective pressures vary (Grant and Grant 2002; Pulido and Berthold 2010), behavioral modification in response to environmental cues (behavioral plasticity) is often likely to be a species’ most effective adaptation in a rapidly changing world (Nussey et al. 2007). However, although behavioral plasticity can allow species to respond appropriately to change (Charmantier et al. 2008), it may also be maladaptive if environments change too rapidly or create novel conditions (Van Buskirk 2012). Understanding how accurately individuals track changes in their environment is vital if we are to predict how they may cope with environmental change now and in the future (Chevin et al. 2010; Van Buskirk 2012).

Brood parasites (“cuckoos”) lay their eggs in the nests of other species, manipulating their hosts into providing care for parasite offspring. Some hosts raise their own young alongside a cuckoo chick, whereas others raise only the cuckoo if it out-competes or forcibly removes its host nest-mates. This high cost of raising parasite young selects for host defenses; some hosts deter parasitism with mobbing attacks of adult cuckoos, by ejecting foreign eggs, or by removing parasitic young from the nest. But, these in turn select for escalating cuckoo offenses to improve their chances of successfully hoodwinking hosts (Rothstein 1990; Davies 2011; Kilner and Langmore 2011).

Interactions between avian hosts and their brood parasites provide a good model for investigating how animals adapt to varying selection pressures. Both cuckoo and host populations vary in relative density, perhaps because they are affected by different ecological factors (e.g., food, conditions during migration and in winter quarters). This leads to heterogeneity in parasitism risk in space and in time, and recent studies have revealed a wide range of host responses to this variation in brood parasitism. When parasites have increased in numbers, or have expanded their geographical range, some hosts have become more likely to reject foreign eggs as a defense (Takasu 1993; Soler and Soler 2000), whereas others continue to accept parasitic eggs (Rothstein 1990). Acceptance might reflect evolutionary lag; egg rejection would pay but there has been insufficient time for it to evolve (Hosoi and Rothstein 2000). Alternatively, there may be strong counter selective pressures against egg rejection so that acceptance of parasitic eggs is always best, despite the costs of parasitism (Rohwer and Spaw 1988; Hoover and Robinson 2007; Krüger 2011; Gloag et al. 2012).

In other cases, brood parasites have declined in numbers, or hosts have expanded into areas free from parasitism. Some hosts have responded by reducing egg rejection (Brooke et al. 1998; Lindholm and Thomas 2000; Lahti 2006; Stokke et al. 2008; Soler et al. 2012), which suggests that this defense is costly for these hosts. However, other hosts always reject foreign eggs, regardless of parasitism risk (Davies and Brooke 1989a; Rothstein 2001; Honza et al. 2004; Peer et al. 2011), so sometimes egg rejection might have insignificant costs (Rothstein 2001). Mobbing defenses also vary among hosts. Although some hosts approach brood parasites and unleash attacks to deter parasitism (reviewed in Feeney et al. 2012), others only approach when the parasite is perceived as a predatory threat (Campobello and Sealy 2011a).

As well as differences among host populations, we also find differences in expression of defenses within populations. For example, individuals that are at greater risk of parasitism are more likely to mob adult parasites (Welbergen and Davies 2009, 2012; Langmore et al. 2012; Thorogood and Davies 2012) and to reject foreign eggs (Soler et al. 2013). In some cases, opportunities for personal and social learning may determine the likelihood and specificity of these defenses (Davies and Welbergen 2009; Campobello and Sealy 2011a, b; Thorogood and Davies 2012; Feeney & Langmore 2013).

This variation in host responses highlights two factors that influence whether populations will respond to environmental change. First, if host defenses are costly, then these should evolve as plastic behaviors, switched on in times of need, and off when costs outweigh the benefits of expression. Second, phenotypic flexibility may allow rapid tracking of environmental change, but it relies on the presence of reliable cues, which may not always be available (Visser et al. 1998). Indeed, brood parasites will be selected to suppress the cues that alert host defenses (Thorogood and Davies 2012; Welbergen and Davies 2012).

Since 1985, we have monitored parasitism by common cuckoos (*Cuculus canorus*) of a population of reed warblers (*Acrocephalus scirpaceus*) on Wicken Fen, Cambridgeshire, U.K. Over the past three decades, we have also measured two host defenses by experiment: mobbing of adult cuckoos (by presentation of cuckoo mounts and models) and egg rejection (by placing model cuckoo eggs into nests). Both defenses are costly for reed warblers. Mobbing an adult cuckoo can reduce the chance that the nest is parasitized (Welbergen and Davies 2009), but close approach is risky because cuckoos resemble hawks (Welbergen and Davies 2011; Thorogood and Davies 2012). Egg rejection may redeem a reed warbler’s reproductive investment, but entails the risk that it rejects its own eggs (Davies et al. 1996). On our study site, both defenses vary in response to fine-scale spatial variation in parasitism risk (mobbing, Welbergen and Davies 2009, 2012; egg rejection, Brooke et al. 1998), and in general, decline through the breeding season (Brooke et al. 1998; also see Soler et al. 2012). This variation is likely to be a direct phenotypic response to parasitism risk, because the sight of cuckoos in the local host neighborhood stimulates both egg rejection (Davies and Brooke 1988; Moksnes et al. 2000; Davies et al. 2003) and mobbing (Davies and Welbergen 2009; Campobello and Sealy 2011b; Thorogood and Davies 2012).

Why should reed warblers show phenotypic flexibility in their antiparasite defenses? Across Europe, reed warblers are patchily distributed along the fringes of reed beds and waterways (Leisler and Schulze-Hagen 2011) and in the United Kingdom, habitat suitable for reed warblers is restricted to small islands of wetland in a sea of agriculture. Populations of cuckoos that specialize on reed warblers are therefore often small on a local scale and prone to stochastic variation and local extinction (Lindholm 1999). This leads to variation in parasitism rates at a given site in successive years (Lindholm 1999) and variation between sites just a few kilometers apart (Brooke et al. 1998). Thus, despite fidelity to a breeding site, an adult reed warbler is likely to encounter variable parasitism risk even over its brief lifetime, and offspring are likely to experience different parasitism risk from their parents, even over the short average dispersal distance of 47 km between natal and breeding sites (Paradis et al. 1998). Perhaps in consequence, reed warbler populations across Europe vary in both mobbing and egg rejection defenses (Lindholm and Thomas 2000; Røskaft et al. 2002; Stokke et al. 2008; Campobello and Sealy 2010), with parasitized populations more likely to defend themselves (Røskaft et al. 2002; Stokke et al. 2008). Whenever defenses are costly, and there is such fine scale spatial and temporal variation in encounter rate with enemies, selection will favor the ability of victims to assess risks and to adjust their defenses accordingly. Flexible defenses would also lead to host populations rapidly tracking any long-term changes in parasitism.

In a previous paper, we reported a decline in cuckoo parasitism and cuckoo egg rejection by reed warblers during the first 12 years of our study (1985–1997; Brooke et al. 1998). During the past 15 years, cuckoos have continued to decline at our study site, in common with other areas of England where the decline overall has been by 56% from 1994 to 2008 (Douglas et al. 2010). Here, we use this variation in parasitism risk experienced by our host population to test whether the host’s first line of defense, mobbing of adult cuckoos, and their second line of defense, egg rejection, respond flexibly to parasitism risk. First, we test whether these two reed warbler defenses have declined over the three decades, in concert with the decline in cuckoos. Second, we test for fine-tuning in host defenses by relating these to parasitism risk during egg laying, when nests are most vulnerable to parasitism (Davies and Brooke 1988). Finally, we test whether our population’s responses to parasitism risk are indicative of a common response across reed warbler populations in Europe. We predicted that the relationship between defenses and parasitism risk in our study population should also explain spatial variation among host populations.

## Methods

### Study Area, Host, and Cuckoo Populations

Our study site is Wicken Fen and the adjacent fenland in Cambridgeshire, United Kingdom (52°18′29″N, 0°16′50″E), where we have studied reed warblers and cuckoos since 1985. Each pair of hosts defends a linear breeding territory of 11–35 m along the reed fringes of waterways (Davies et al. 2003). Cuckoos are harder to monitor, but some lay in well-defined territories and from individual differences in their eggs we estimated how many female cuckoos were active on the fen each year (Davies and Brooke 1988). Field effort each year varied, but we always monitored all nests in two areas (Wicken Lode and Wicken Sedge Fen east of Drainer’s dyke) to provide an index of reed warbler abundance. For more details see Brooke et al. (1998).

### Measuring Host Mobbing of Adult Cuckoos

We assessed defensive mobbing on the day of clutch completion in 13 of the years from 1985 to 2012. We presented cuckoo mounts (1985–2008) or wooden models of cuckoos (2009–2012) at host nests, in direct contact with the nest rim and recorded the number of bill snaps and rasp calls within 5 minutes after the arrival of the first reed warbler to within 1 m of the nest. Bill snaps and rasp calls are correlated with close approach, threat postures and direct attack of cuckoo models and so are good measures of mobbing (Welbergen and Davies 2008). Responses did not differ between mounts (*N* = 4) or wooden models (*N* = 2; Welbergen and Davies 2008) or between the different model types (Thorogood and Davies 2012). Observations of color-ringed birds showed that those first to arrive were invariably the nest owners (Davies et al. 2003). We also measured mobbing response to presentations of a nest predator (Eurasian jay, *Garrulus glandarius*) and a predator of adults (Eurasian sparrowhawk, *Accipiter nisus*) to control for possible changes in response to enemies overall. Each pair was tested only once in a given season, but a small proportion of pairs may have been resampled in other years. However, annual survival rates of reed warblers are low (Thaxter et al. 2006), and we sampled less than 20% of pairs in our study population in any 1 year. Therefore, this replication is unlikely to influence our results.

### Measuring Host Rejection of Eggs

We assessed egg rejection in 1985 to 1986, 1997, and 2012. We placed one model cuckoo egg in a nest during the laying period, mostly on the day the fourth egg was laid (most clutches were of four eggs). The eggs, of the same size and mass as real cuckoo eggs, were made of resin and painted with acrylic paints. Three types were used to represent three different host-races of cuckoo (Brooke et al. 1998):

1. Pied wagtail host-race: a pale gray background, lightly speckled with brown spots.
2. Redstart host-race: an immaculate pale blue egg.
3. Reed warbler host-race: a pale green background, heavily speckled with green spots.

The first two types were clearly different from the reed warbler’s own eggs (“nonmimetic”), whereas the third resembled the warbler’s eggs (“mimetic”). These model eggs do not, of course, present an entirely realistic cuckoo egg because they cannot be punctured and may not have all the colors as perceived by a bird’s eye. Nevertheless, reed warblers are more likely to accept “mimetic” model eggs (Davies and Brooke 1988) and host rejection of “nonmimetic” model eggs predicts the degree of cuckoo egg mimicry across different cuckoo host-races as modeled by bird vision (Stoddard and Stevens 2011). Therefore, response to these model eggs provides a valid, standard measure of host rejection across the years. Mimetic models control for other factors that might influence egg rejection or nest desertion. Each reed warbler pair was tested only once per season.

At some nests in 1985, we removed a host egg when we placed a model egg in the nest, as real cuckoos do. However, host egg removal had no effect on host rejection of model eggs (Davies and Brooke 1988), so in subsequent years we simply added the model egg to the clutch. Nests were then monitored every other day. The model egg was scored as “rejected” if it had disappeared from the nest by ejection, or if the clutch had been deserted, and scored as “accepted” if it was still present and being incubated 6 days after clutch completion, the same criterion as used in our first study (Davies and Brooke 1988).

Most pairs were tested with either a mobbing trial or an egg rejection trial. However, a sample of pairs was presented with a model or mount of an adult cuckoo (5 min at the nest after first approach to 1 m) before a mimetic model egg was added to the clutch, to test whether the sight of a cuckoo at the nest stimulated egg rejection.

### Comparisons with Other Populations

We compared our study population’s response to published data on mobbing behavior and egg rejection from other sites for which an estimate of the population’s parasitism rate and sample size was available. We first estimated a line of best fit using a binomial generalized linear model (GLM) of the ratio of nests where the defense was recorded, and then used *Z*-tests to compare the slope of these lines with the ones we estimated for our study population (see Table S3 for details of populations). These published data did not all use identical experimental methods to us, so the fit of these data to ours is likely to be an underestimate of how closely our study population’s response to parasitism predicts others.

### Statistical Analyses

To test for changes in cuckoo and host populations, we used linear models with significance assessed against the *F*-distribution. For changes in data expressed as a proportion of the population (parasitism, mobbing, and rejection propensity), we used binomial GLM with the χ^2^ distribution, or Fisher’s exact tests to analyze differences in overall proportions. To investigate how host defenses (does not mob/mobs, accepts/rejects) change in response to parasitism rate during the host’s laying period, we measured parasitism rate during a 3-day period, starting with the laying of the first egg, as this is when clutches are most vulnerable to parasitism (Davies and Brooke 1988), and we used GLMs with a binomial error and logit link function (or quasi-binomial errors if overdispersed) to analyze these data. All analyses were conducted in R 2.15.1 (R Core Team 2012).

## Results

### On Wicken Fen

**Figure 1:**
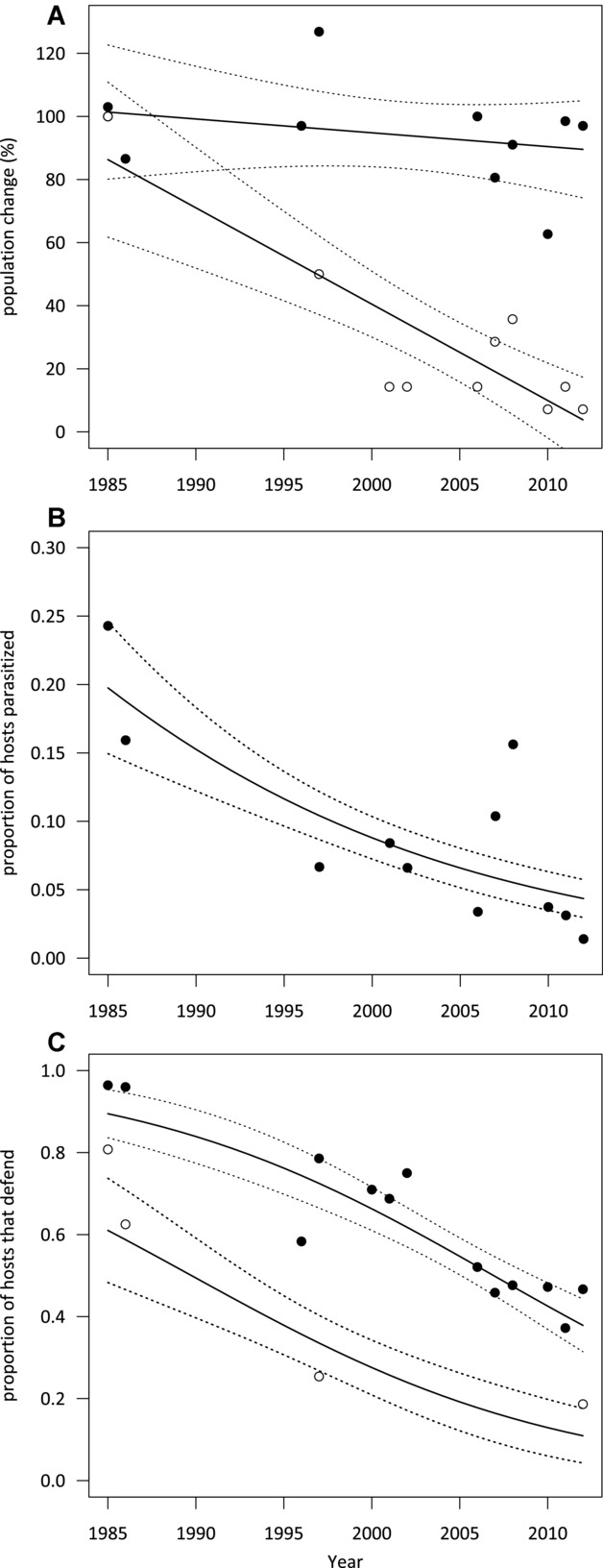
Changes in (A) population sizes (1985 scored as 100%) of reed warbler hosts (black circles) and female cuckoos (white circles); (B) parasitism rate; and (C) host defenses (mobbing of adult cuckoos, black circles, and rejection of non-mimetic eggs, white circles) at Wicken Fen, Cambridgeshire, U.K., from 1985–2012. Lines of best fit (± SE) come from linear regressions (A), or binomial generalized linear models (B, C). See Table S1 for raw data.

Although reed warbler abundance has not changed during the last three decades (*F*_(1,8)_ = 0.64, *P* = 0.45), cuckoos on Wicken Fen have not fared well (Fig. 1A). The number of females has drastically declined (*F*_(1,8)_ = 25.8, *P* = 0.001), and consequently so too have parasitism rates (χ^2^ = 42.8, *P* < 0.001; Fig. 1B). Reed warbler defenses have tracked this decline in parasitism. By 2012, less than half of the population mobbed cuckoos compared to 96% in the 1980s (χ^2^ = 55.2, *P* < 0.001; Fig. 1C). Rejection of nonmimetic eggs also declined, from 73.8% in the 1980s to 18.6% in 2012 (χ^2^ = 28.63, *P* < 0.001; Fig. 1C, Table 1). As parasitism rates declined, we found that the presentation of an adult cuckoo model at the nest no longer increased rejection of mimetic eggs (Table 1, Fisher’s Exact *P* = 1 for both 1997 and 2012), as it did in earlier years when cuckoos were most abundant (1985–1986: *P* = 0.004).

**Table 1:** Change in rejection rates of nonmimetic (redstart type or pied wagtail type) or mimetic (reed warbler type) model cuckoo eggs by reed warblers. Some mimetic eggs were inserted into nests after an adult cuckoo model was shown at the nest. Significant differences in rejection of nonmimetic eggs are tested using a binomial generalized model and a χ^2^ distribution (df = 1) and responses to mimetic eggs are compared using Fisher’s exact tests. There was no difference in rejection rate of the two nonmimetic egg models (redstart or pied wagtail types, year*nonmimetic type: χ^2^_(2)_ = 0.45, *P* = 0.80) so these were combined for comparisons among years

	Number of cuckoo eggs rejected/total				
	Nonmimetic eggs			Mimetic eggs	
Year	Redstart-type	Pied wagtail-type	Total	No adult cuckoo	After adult cuckoo
1985–1986	18/26	13/16	31/42	1/25	9/23
	(69.2%)	(81.3%)	(73.8%)	(4.0%)	(39.1%)
1997	14/57	18/69	32/126	6/39	5/30
	(24.6%)	(26.1%)	(25.4%)	(15.4%)	(16.7%)
2012	5/30	6/29	11/59	3/26	1/14
	(16.7%)	(20.7%)	(18.6%)	(11.5%)	(7.1%)
Overall effect of year			*P*<0.001	*P*=0.44	*P*=0.068
Change from 1985–1986 to 1997			*P*<0.001	*P*=0.23	*P*=0.12
Change from 1997–2012			*P*=0.31	*P*=0.73	*P*=0.65

These declines in defenses are not due to changes in overall aggression of reed warblers, or because of other factors that may influence desertion or re-nesting. Reed warblers mobbed predators at the nest at similar rates across the time period (jay, 1987–1988: 6/12 pairs mobbed, 2002: 5/13; sparrowhawk, 1987–1988: 3/11, 2006–2008: 6/24; data from Duckworth 1991; Davies et al. 2003; Welbergen and Davies 2012), and mimetic model eggs (our control) were rejected very rarely, and there was no change over time (Table 1). Neither has egg rejection declined because its cost has changed. Reed warblers were just as likely to reject a nonmimetic model egg by ejecting it from the nest as in previous periods (ejection of a cuckoo egg is less costly than desertion as it does not require rebuilding of a nest; 1985–1986: 17/30 rejections were by ejection, 1997: 11/33, 2012: 4/11, Fisher’s exact test, *P* = 0.16), and the proportion of host eggs that were damaged or removed when there was a model egg in the nest did not differ among years (1985–1986: 8/17 ejections involved at least one host egg, 1997: 9/17, 2012: 1/5, Fisher’s exact *P* = 0.51).

**Figure 2:**
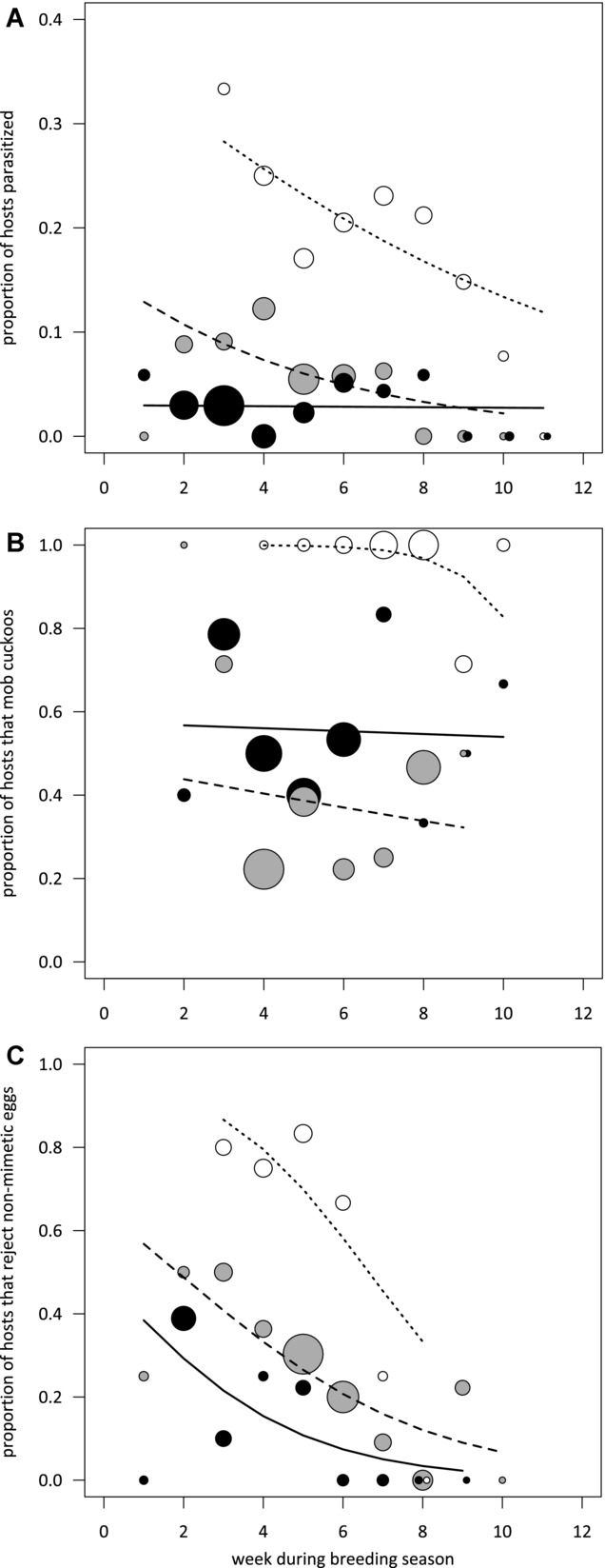
Seasonal changes on Wicken Fen in the proportion of reed warblers that (A) were parasitized by cuckoos; (B) mobbed an adult cuckoo model at the nest; and (C) rejected nonmimetic model eggs, with week 1 beginning May 14. White circles (with short dashed lines) represent data from 1985 to 1986, gray circles (with long-dashed lines) represent data from 1996 to 1997, and black circles (with solid lines) represent data from 2010 to 2012. Circle diameters are relative to sample size and an x-axis jitter of 0.1 was applied to display overlapping data.

Previous analyses of our data from 1985 to 1986 and 1997 found that reed warblers were less likely to reject nonmimetic eggs as the breeding season progressed, perhaps because parasitism also declined through the season (Brooke et al. 1998). By looking within season, across years, we found that parasitism rates in 2010–2012 were so low that any seasonal variation was no longer statistically detectable (Fig. 2A). We did not detect any seasonal declines in mobbing behavior during any of these same periods (1985–1986, 1996–1997, 2010–2012; year*week during season, χ^2^ = 2.50, *P* = 0.29; week during season, χ^2^ = 0.54, *P* = 0.46; Fig. 2B). However, reed warblers were less likely to reject nonmimetic eggs as the breeding season progressed (week during season, slope = −0.37 ± 0.09, χ^2^ = 17.57, *P* < 0.0001; Fig. 2C), and this effect did not differ between years (year*week during season, χ^2^ = 0.52, *P* = 0.77).

From these data, however, we cannot know if these seasonal declines are in response to changes in parasitism, or because of other life-history variables. For example, early nesting birds are likely to be older, with more experience of their own eggs (Lotem et al. 1995), and earlier in the season there is also a greater chance of re-nesting, so the costs of deserting a clutch with a possible cuckoo egg are lower. This also means that repeatedly testing individuals’ responses to cuckoos is not straightforward, particularly as repeated presentation of the models themselves may inflate the birds’ perception of parasitism risk (Samaš et al. 2011). Therefore, we next looked at variation among reed warblers in their response to parasitism risk at the time of laying, as a way of estimating how individuals may respond to varying parasitism risk within their lifetime.

**Figure 3:**
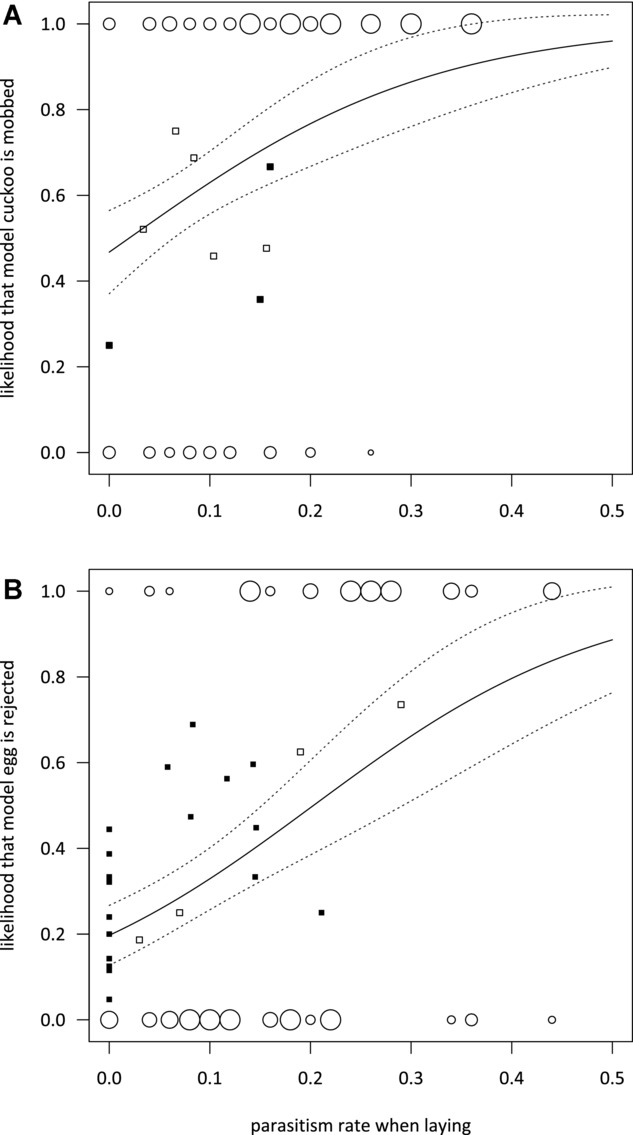
Variation in defenses by reed warblers on Wicken Fen in response to parasitism rate at the time of laying, as predicted by a generalized linear model with binomial errors (solid line, dashed lines ± SE) of (A) mobbing of adult cuckoo models at the nest (not mobbed (0) or mob (1), *n* = 205 nests from 1985 to 1986, 1996 to 1997, 2010 to 2011) and (B) acceptance (0) or rejection (1) of nonmimetic model eggs (*n* = 201 nests from 1985 to 1986, 1997, 2012). The relative sizes of pairs of data points (open circles) show the proportion of birds that did or did not defend themselves. Also plotted are the data for geographic variation in reed warbler defenses at various populations across Europe: black squares represent population-level estimates of mobbing or egg rejection at varying parasitism rates from other study sites (see Table S3 for details). Population-level data from different years on Wicken Fen were not used to investigate fit of other populations, but are presented here for comparison (white squares).

By pooling data from the years when experiments were performed, we found that reed warblers were more likely to mob an adult cuckoo model at their nest (Fig. 3A), and more likely to reject a nonmimetic egg (Fig. 3B), when the parasitism rate of the population at the time of laying was higher (slope for mobbing = 6.61 ± 1.83, χ^2^ = 15.01, *P* < 0.001; slope for egg rejection = 6.92 ± 1.48, χ^2^ = 25.57, *P* < 0.001). We considered whether these responses were driven by seasonal differences (week during season weakly, but significantly correlated with parasitism rates; *r*^2^ = 0.17, *P* = 0.006). However, our results remained unchanged if seasonal effects were included in the models (mobbing: week during season, χ^2^ = 3.54, *P* = 0.058, parasitism during laying, χ^2^ = 15.17, *P* < 0.001; rejection: week during season, χ^2^ = 10.30, *P* = 0.001, parasitism during laying, χ^2^ = 22.23, *P* < 0.001).

A potential problem with these analyses is that we did not have sufficient replication of responses to variable parasitism rates in each of the three time periods (1985–1986, 1996–1997, 2010–2012), so the response curves we calculated could simply be due to overall differences among years (Fig. 1). Therefore, we also restricted our analyses to one time period where we had a large range of parasitism rates (1985–1986, see Fig. S2), but found that relationships between parasitism rate during laying and an individual’s probability of defending itself remained similar (although no longer statistically significant at α = 0.05; slope for rejection = 4.24 ± 2.76, χ^2^ = 2.52, *P* = 0.11, cases of pairs not mobbing (2/53) were too infrequent to analyze).

### Comparison with Other Populations Across Europe

**Figure 4:**
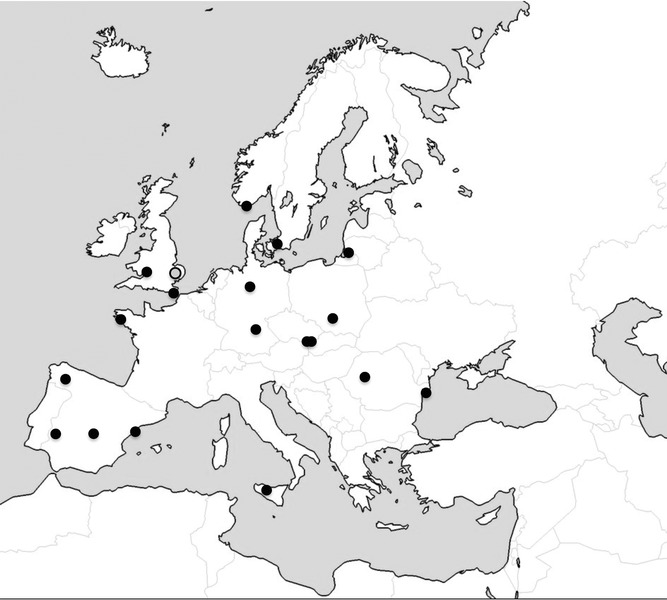
Data from Wicken Fen (gray circle) were compared to data from reed warbler populations across Europe (black circles). Details of these studies are given in Table S3. Map adapted from http://neethis.deviantart.com.

Finally, we tested whether the fine-scale modulation in defenses in response to local parasitism risk on Wicken Fen could explain variation in defenses across different, geographically distant, populations (Fig. 4, also see Table S3). Comparing other reed warbler populations (black squares in Fig. 3), egg rejection increased with overall parasitism risk (Fig. 3B, slope = 7.07 ± 2.69, χ^2^ = 25.53, *P* = 0.008), and the line of best fit from these other populations did not differ from the slope calculated from our Wicken Fen data (compared to model controlling for seasonal effects: *Z* = 0.14, *P* = 0.44). Data from these other populations were collected on a different scale to our Wicken Fen data (proportions of populations parasitized and rejecting model eggs over an entire season), but if we ignored these differences and converted our Wicken Fen egg rejection data to compare slopes directly, then again there was no difference (population*parasitism rate for season: χ^2^ = 0.01, *P* = 0.91, parasitism rate: slope = 7.04 ± 1.02, χ^2^ = 51.82, *P* < 0.0001).

We do not have sufficient data on mobbing defenses by other populations of reed warblers to test these similarities for defensive mobbing (Fig. 3A). However, the responses of Wicken Fen reed warblers predict host defenses to reach 99% prevalence (encompassed by upper standard error) once cuckoos parasitize at least 48% (mobbing) or 62% (egg rejection) of a host population.

## Discussion

### Declining Cuckoos and Host Defenses

The causes of the marked decline in cuckoos in Britain are not known. Common cuckoos have several host-races, each specializing on one particular host (Brooke and Davies 1988; Gibbs et al. 2000; Fossøy et al. 2011), but there is no evidence that a decline in any British host is a major contributor to the cuckoo decline (Douglas et al. 2010). Indeed, over the United Kingdom as a whole reed warblers have increased by 26% from 1994 to 2008 (Douglas et al. 2010), and on Wicken Fen our censuses suggest that their numbers have remained steady since 1985. So the decline in parasitism on our study site is due to the decline in cuckoos, and the decline in cuckoos has not led to a detectable increase in hosts. Climate change has led to earlier breeding in some hosts, so there could be reduced host availability if cuckoo arrival time in spring had not kept pace with these changes (Saino et al. 2009). However, there is no evidence that a mismatch between host and cuckoo breeding phenology might explain the cuckoo decline in Britain (Douglas et al. 2010). In some cases, increased host defenses can cause declines in cuckoo populations (Nakamura et al. 1998; Rothstein 2001). However, in our study the decline in cuckoos was accompanied by a *decline* in host defenses. More likely causes for the cuckoo’s precipitous decline are less food for adult cuckoos in the breeding season (mainly caterpillars; Conrad et al. 2006), or deteriorating conditions on migration or in sub-Saharan wintering sites (Ockendon et al. 2012).

Our results show that, whatever the causes of the cuckoo decline, since 1985 reed warblers have rapidly tracked their declining risk of parasitism on Wicken Fen with a decline in both their first line of defense, mobbing adult cuckoos, and in egg rejection. There are at least three possible reasons for this decline in host defenses: (i) individuals show “fixed” expression of defenses and the proportion of different genotypes (“acceptors” vs. “rejectors” or “nonmobbers” vs. “mobbers”) has changed due to rapid selection or migration among populations; (ii) individuals vary in how plastic their defenses are in response to parasitism risk (Dingemanse et al. 2010), with more responsive individuals no longer favored by selection; or (iii) there is a ‘generalized norm of reaction’ (Sarkar and Fuller 2003) which describes how individuals across populations respond in similar ways to variation in an environmental cue.

### Genetic Change Versus Plasticity

Some changes in prey or host defenses involve genetic change. For example, male guppies (*Poecilia reticulata*) evolve to be more brightly colored under relaxed predation (Endler 1980). Egg patterns in cuckoo hosts also evolve in response to changing parasitism pressure, becoming less variable between individual females under reduced parasitism (Lahti 2005), and more individually distinctive in response to continuing parasitism, leading to a signature-forgery arms race between hosts and parasites (Spottiswoode and Stevens 2012). These changes in egg patterns can evolve over a few decades and they influence host egg rejection; less distinct individual egg patterns compromises a host’s ability to detect a foreign egg (Lahti 2006), whereas more distinctive markings enhance egg discrimination (Spottiswoode and Stevens 2010).

It is unlikely, however, that the changes we have detected at Wicken Fen are because of relative changes in the abundance of polyphenic “defensive” or “accepting” genotypes. Previous analysis favored phenotypic flexibility as the likely cause of the decline in egg rejection during the first 12 years of our study (1985–1997) because calculations using the genetic model of Takasu et al. (1993) showed that the decline was too rapid to reflect only genetic change (Brooke et al. 1998). More convincingly perhaps, the magnitudes of the changes in defenses we have detected over three decades are within the range of individual variation expressed over just 1 year in response to variation in local parasitism risk. We also know from experiments that individual reed warblers modify their defenses. The extent of these changes varies among individuals, but overall the increase in the proportion of birds that mob cuckoos in response to social information is similar to the changes we have detected here across years (e.g., 30% more pairs mob cuckoos after observing their neighbors mob models; Davies and Welbergen 2009; Thorogood and Davies 2012). Our experiments also show that the sight of a cuckoo at the nest sometimes modifies egg rejection (increasing the number of pairs who reject by 35% in 1985). In the closely related great reed warbler (*Acrocephalus arundinaceus*, another common cuckoo host), experience with nonmimetic eggs decreases rejection thresholds (Hauber et al. 2006), but these experiments are yet to be done with reed warblers.

### From Individual to Species’ Responses

The response curve that we calculated for egg rejection in our population explained variation across other populations reasonably well, especially given that not all studies used precisely the same experimental methods (see Table S3). This suggests that reed warblers might demonstrate a similar, flexible response to parasitism risk across their range. Interestingly, our response curves (Fig. 3) also predict some individuals in a population to always exhibit some defense toward cuckoos, even when parasitism risk is nil. Indeed, at sites not parasitized by cuckoos, a small proportion of reed warblers still attack adult cuckoos or reject foreign eggs (Lindholm and Thomas 2000; Stokke et al. 2008; Welbergen and Davies 2012), including populations at the frontier of their range (Avilés et al. 2006; Stokke et al. 2008; Leisler and Schulze-Hagen 2011).

Why does relaxed selection not lead to loss of plasticity? Although the expression of host defenses is costly, maintaining the physiological mechanisms modulating plasticity (e.g., sensory processes for detecting cuckoo activity) may not be, in which case adaptive divergence is unlikely to be favored (Crispo 2008). In addition, models show that even small amounts of gene flow can impede genetic specialization of populations to different environmental optima, therefore maintaining the genetic architecture necessary for plastic responses (Sultan and Spencer 2002; Crispo 2008; Thibert-Plante and Hendry 2011). Genetic differentiation of reed warblers across Eurasia is low (Procházka et al. 2011), so perhaps dispersal maintains plasticity in unparasitized populations (Soler and Soler 2000).

Why do not all brood parasite hosts adopt plastic defenses? Although common cuckoos may parasitize multiple hosts, within habitats females tend to favor only one species (Fossøy et al. 2011). This means that cues of parasite activity are potentially more reliable indicators of parasitism risk to cuckoo hosts than for hosts of more generalist parasites, such as brown-headed cowbirds (*Molothrus ater*). Here, monitoring parasitism risk is likely to be a harder task because parasite abundance alone will not necessarily indicate parasitism risk to any one particular host species. Although in general, cowbird hosts may show stronger defenses when sympatric than allopatric with parasites (Briskie et al. 1992), their defenses may consequently be less fine-tuned than the reed warblers’ responses we show here.

### Consequences of Host Plasticity for Cuckoos

Behavioral plasticity has important implications for the evolutionary trajectories of parasites too (Agrawal 2001). The lag between plastic expression of a trait and its optimum phenotype under environmental change can determine whether populations persist or go extinct (Chevin et al. 2010), and this lag may also affect interacting species. For example, if hosts accurately adjust their defenses in response to changes in parasitism, then any increase in the cuckoo population unrelated to breeding success (e.g., improved conditions over winter) will be met by increased host defenses. This would stymie cuckoos’ reproductive success and potentially lead to localized extinction (Lindholm 1999). If a host retains defenses under reduced parasitism, then the only option for the parasite is to become more specialized on an old host, or continually change to new hosts. If, however, a host loses defenses under reduced parasitism, then there will be a geographic mosaic of coevolution in space (Martín-Gálvez et al. 2007) and unparasitized host populations will be vulnerable to future parasitism (co-evolutionary cycles; Davies and Brooke 1989b; Marchetti 1992; Rothstein 2001).

The variation in responses toward cuckoos and their eggs that we have identified here, within and across populations, is most likely explained by individuals adjusting their defenses in response to parasitism risk. As cuckoos have declined on Wicken Fen, not only are defenses less likely to be triggered by the sight of a cuckoo near the nest, but an increasing proportion of hosts is likely to be naïve about cuckoos as a threat. This may explain why reed warblers are now less inclined to reject parasitic eggs, even when an adult cuckoo is presented at the nest. Previous experiments (Davies and Welbergen 2009; Campobello and Sealy 2011b; Thorogood and Davies 2012) have demonstrated that information about cuckoos is transmitted socially, resulting in escalated mobbing responses. Perhaps social information is key for maintaining flexibility of egg rejection too. As there is spatial and temporal variation in host defenses within reed warbler populations (e.g., older birds have more experience; some birds are more likely to be parasitized than others), then this heterogeneity of experience may help to rapidly reignite expression of defenses if parasite numbers increase again. Understanding how hosts use information to make these decisions is therefore a key question for future research.

**Associate Editor: B. Lyon**

## Supporting Information

**S1:** Data table for Figure 1 **S2:** Distribution of data for Figure 3 **S3:** Data table and references for Figures 3 & 4 **Table S1**. Changes in cuckoo and reed warbler populations (from cuckoo egg observations and standardized nest searching effort), parasitism rate (proportion of all nests found that contained cuckoo eggs), and expression of hosts’ first line of defense (mobbing an adult cuckoo) at Wicken Fen, Cambridgeshire, U.K., from 1985 to 2012. **Figure S2**. Distribution of parasitism rates during laying in each year we conducted experiments testing (a) mobbing responses and (b) rejection of nonmimetic eggs at reed warbler nests. **Table S3**. Parasitism rates and proportion of hosts who (a) mob taxidermy adult cuckoo models or (b) reject non-mimetic eggs in other reed warbler populations across Europe. **References for Table S3** 

## References

[b1] Agrawal AA (2001). Phenotypic plasticity in the interactions and evolution of species. Science.

[b2] Avilés JM, Stokke BG, Moksnes A, Røskaft E, Asmul M, Møller AP (2006). Rapid increase in cuckoo egg matching in a recently parasitized reed warbler population. J. Evol. Biol.

[b3] Briskie JV, Sealy SG, Hobson KA (1992). Behavioral defenses against avian brood parasitism in sympatric and allopatric host populations. Evolution.

[b4] Brooke MdeL, Davies NB (1988). Egg mimicry by cuckoos *Cuculus canorus* in relation to discrimination by hosts. Nature.

[b5] Brooke MdeL, Davies NB, Noble DG (1998). Rapid decline of host defences in response to reduced cuckoo parasitism: behavioural flexibility of reed warblers in a changing world. Proc. R. Soc. Lond. B.

[b6] Campobello D, Sealy SG (2010). Enemy recognition of reed warblers (*Acrocephalus scirpaceus*): threats and reproductive value act independently in nest defence modulation. Ethology.

[b7] Campobello D, Sealy SG (2011a). Nest defence against avian brood parasites is promoted by egg-removal events in a cowbird-host system. Anim. Behav.

[b8] Campobello D, Sealy SG (2011b). Use of social over personal information enhances nest defense against avian brood parasitism. Behav. Ecol.

[b9] Charmantier A, McCleery RH, Cole LR, Perrins C, Kruuk LEB, Sheldon BC (2008). Adaptive phenotypic plasticity in response to climate change in a wild bird population. Science.

[b10] Chevin L-M, Lande R, Mace GM (2010). Adaptation, plasticity, and extinction in a changing environment: towards a predictive theory. PLoS Biol.

[b11] Conrad KF, Warren MS, Fox R, Parsons MS, Woiwood IP (2006). Rapid declines of common, widespread British moths provide evidence of an insect biodiversity crisis. Biol. Conserv.

[b12] Crispo E (2008). Modifying effects of phenotypic plasticity on interactions among natural selection, adaptation and gene flow. J. Evol. Biol.

[b13] Davies NB (2011). Cuckoo adaptations: trickery and tuning. J. Zool.

[b14] Davies NB, Brooke MdeL (1988). Cuckoos versus reed warblers: adaptations and counteradaptations. Anim. Behav.

[b15] Davies NB, Brooke MdeL (1989a). An experimental study of co-evolution between the cuckoo, *Cuculus canorus*, and its hosts. I. Host egg discrimination. J. Anim. Ecol.

[b16] Davies NB, Brooke MdeL (1989b). An experimental study of co-evolution between the cuckoo, *Cuculus canorus*, and its hosts. II. Host egg markings, chick discrimination and general discussion. J. Anim. Ecol.

[b19] Davies NB, Welbergen JA (2009). Social transmission of a host defense against cuckoo parasitism. Science.

[b17] Davies NB, Brooke MdeL, Kacelnik A (1996). Recognition errors and probability of parasitism determine whether reed warblers should accept or reject mimetic cuckoo eggs. Proc. R. Soc. Lond. B.

[b18] Davies NB, Butchart SHM, Burke TA, Chaline N, Stewart IRK (2003). Reed warblers guard against cuckoos and cuckoldry. Anim. Behav.

[b20] Dingemanse NJ, Kazem AJN, Réale D, Wright J (2010). Behavioural reaction norms: animal personality meets individual plasticity. Trends Ecol. Evol.

[b21] Douglas DJT, Newson SE, Leech DI, Noble DG, Robinson RA (2010). How important are climate-induced changes in host availability for population processes in an obligate brood parasite, the European cuckoo?. Oikos.

[b22] Duckworth JW (1991). Responses of breeding Reed Warblers *Acrocephalus scirpaceus* to mounts of Sparrowhawk *Accipiter nisus*, Cuckoo *Cuculus canorus* and Jay *Garrulus glandarius*. Ibis.

[b23] Endler JA (1980). Natural selection on color patterns in *Poecilia reticulata*. Evolution.

[b25] Feeney WE, Langmore NE (2013). Social learning of a brood parasite by its host. Biol. Lett.

[b24] Feeney WE, Welbergen JA, Langmore NE (2012). The frontline of avian brood parasite–host coevolution. Anim. Behav.

[b26] Fossøy F, Antonov A, Moksnes A, Røskaft E, Vikan JR, Møller AP, Shykoff JA, Stokke BG (2011). Genetic differentiation among sympatric cuckoo host races: males matter. Proc. R. Soc. B.

[b27] Gibbs HL, Sorenson MD, Marchetti K, Brooke MdeL, Davies NB, Nakamura H (2000). Genetic evidence for female host-specific races of the common cuckoo. Nature.

[b28] Gloag R, Fiorini VD, Reboreda JC, Kacelnik A (2012). Brood parasite eggs enhance egg survivorship in a multiply parasitized host. Proc. R. Soc. B.

[b29] Grant PR, Grant BR (2002). Unpredictable evolution in a 30-year study of Darwin’s finches. Science.

[b30] Hauber ME, Moskát C, Bán M (2006). Experimental shift in hosts’ acceptance threshold of inaccurate-mimic brood parasite eggs. Biol. Lett.

[b31] Honza M, Grim T, Čapek M, Moksnes A, Røskaft E (2004). Nest defence, enemy recognition and nest inspection behaviour of experimentally parasitized Reed Warblers *Acrocephalus scirpaceus*. Bird Study.

[b32] Hoover JP, Robinson SK (2007). Retaliatory mafia behavior by a parasitic cowbird favors host acceptance of parasitic eggs. Proc. Natl. Acad. Sci. USA.

[b33] Hosoi SA, Rothstein SI (2000). Nest desertion and cowbird parasitism: evidence for evolved responses and evolutionary lag. Anim. Behav.

[b34] Kilner RM, Langmore NE (2011). Cuckoos versus hosts in insects and birds: adaptations, counter-adaptations and outcomes. Biol. Rev. Camb. Philos. Soc.

[b35] Krüger O (2011). Brood parasitism selects for no defence in a cuckoo host. Proc. R. Soc. Lond. B.

[b36] Lahti DC (2005). Evolution of bird eggs in the absence of cuckoo parasitism. Proc. Natl. Acad. Sci. USA.

[b37] Lahti DC (2006). Persistence of egg recognition in the absence of cuckoo brood parasitism: pattern and mechanism. Evolution.

[b38] Langmore NE, Feeney WE, Crowe-Riddell J, Luan H, Louwrens KM, Cockburn A (2012). Learned recognition of brood parasitic cuckoos in the superb fairy-wren *Malurus cyaneus*. Behav. Ecol.

[b39] Leisler B, Schulze-Hagen K (2011). The reed warblers.

[b40] Lindholm AK (1999). Brood parasitism by the cuckoo on patchy reed warbler populations in Britain. J. Anim. Ecol.

[b41] Lindholm AK, Thomas RJ (2000). Differences between populations of reed warblers in defences against brood parasitism. Behaviour.

[b42] Lotem A, Nakamura H, Zahavi A (1995). Constraints on egg discrimination and cuckoo–host co-evolution. Anim. Behav.

[b43] Marchetti K (1992). Costs to host defence and the persistence of parasitic cuckoos. Proc. R. Soc. Lond. B.

[b44] Martín-Gálvez D, Soler JJ, Martínez JG, Krupa AP, Soler M, Burke T (2007). Cuckoo parasitism and productivity in different magpie subpopulations predict frequencies of the 457bp allele: a mosaic of coevolution at a small geographic scale. Evolution.

[b45] Moksnes A, Hagen L, Honza M (2000). Common cuckoo *Cuculus canorus* and host behaviour at reed warbler *Acrocephalus scirpaceus* nests. Ibis.

[b46] Nakamura H, Kubota S, Rothstein SI, Robinson SK, Suzuki R (1998). Coevolution between the common cuckoo and its major hosts in Japan: stable versus dynamic specialization in hosts. Parasitic birds and their hosts: studies in coevolution.

[b47] Nussey DH, Wilson AJ, Brommer JE (2007). The evolutionary ecology of individual phenotypic plasticity in wild populations. J. Evol. Biol.

[b48] Ockendon N, Hewson CM, Johnston A, Atkinson PW (2012). Declines in British-breeding populations of Afro-Palaearctic migrant birds are linked to bioclimatic wintering zone in Africa, possibly via constraints on arrival time advancement. Bird Study.

[b49] Paradis E, Baillie SR, Sutherland WJ, Gregory RD (1998). Patterns of natal and breeding dispersal in birds. J. Anim. Ecol.

[b50] Peer BD, Kuehn MJ, Rothstein SI, Fleischer RC (2011). Persistence of host defence behaviour in the absence of avian brood parasitism. Biol. Lett.

[b51] Procházka P, Stokke BG, Jensen H, Fainová D, Bellinvia E, Fossøy F, Vikan JR, Bryja J, Soler M (2011). Low genetic differentiation among reed warbler *Acrocephalus scirpaceus* populations across Europe. J. Avian Biol.

[b52] Pulido F, Berthold P (2010). Current selection for lower migratory activity will drive the evolution of residency in a migratory bird population. Proc. Natl. Acad. Sci. USA.

[b53] R Core Team (2012). R: A language and environment for statistical computing.

[b54] Rohwer S, Spaw CD (1988). Evolutionary lag versus bill-size constraints: a comparative study of the acceptance of cowbird eggs by old hosts. Evol. Ecol.

[b55] Røskaft E, Moksnes A, Stokke BG, Bicik V, Moskat C (2002). Aggression to dummy cuckoos by potential European cuckoo hosts. Behaviour.

[b56] Rothstein SI (1990). A model system for coevolution: avian brood parasitism. Annu. Rev. Ecol. Syst.

[b57] Rothstein SI (2001). Relic behaviours, coevolution and the retention versus loss of host defences after episodes of avian brood parasitism. Anim. Behav.

[b58] Saino N, Rubolini D, Lehikoinen E, Sokolov LV, Bonisoli-Alquati A, Ambrosini R, Boncoraglio G, Møller AP (2009). Climate change effects on migration phenology may mismatch brood parasitic cuckoos and their hosts. Biol. Lett.

[b59] Samaš P, Hauber ME, Cassey P, Grim T (2011). Repeatability of foreign egg rejection: testing the assumptions of co-evolutionary theory. Ethology.

[b60] Sarkar S, Fuller T (2003). Generalized norms of reaction for ecological developmental biology. Evol. Dev.

[b61] Soler M, Soler J (2000). Brood-parasite interactions between great spotted cuckoos and magpies: a model system for studying coevolutionary relationships. Oecologia.

[b62] Soler M, Martín-Vivaldi M, Fernández-Morante J (2012). Conditional response by hosts to parasitic eggs: the extreme case of the rufous-tailed scrub robin. Anim. Behav.

[b63] Soler JJ, Martín-Gálvez D, de Neve L, Soler M (2013). Brood parasitism correlates with the strength of spatial autocorrelation of life history and defensive traits in magpies. Ecology.

[b64] Spottiswoode CN, Stevens M (2010). Visual modeling shows that avian host parents use multiple visual cues in rejecting parasitic eggs. Proc. Natl. Acad. Sci. USA.

[b65] Spottiswoode CN, Stevens M (2012). Host-parasite arms races and rapid changes in bird egg appearance. Am. Nat.

[b66] Stoddard MC, Stevens M (2011). Avian vision and the evolution of egg color mimicry in the common cuckoo. Evolution.

[b67] Stokke BG, Hafstad I, Rudolfsen G, Moksnes A, Møller AP, Røskaft E, Soler M (2008). Predictors of resistance to brood parasitism within and among reed warbler populations. Behav. Ecol.

[b68] Sultan S, Spencer H (2002). Metapopulation structure favors plasticity over local adaptation. Am. Nat.

[b69] Takasu F, Kawasaki K, Nakamura H, Cohen JE, Shigesada N (1993). Modelling the population dynamics of a cuckoo-host association and the evolution of host defences. Am. Nat.

[b70] Thaxter CB, Redfern CPF, Bevan RM (2006). Survival rates of adult reed warblers *Acrocephalus scirpaceus* at a northern and southern site in England. Ringing Migration.

[b71] Thibert-Plante X, Hendry AP (2011). The consequences of phenotypic plasticity for ecological speciation. J. Evol. Biol.

[b72] Thorogood R, Davies NB (2012). Cuckoos combat socially transmitted defenses of reed warbler hosts with a plumage polymorphism. Science.

[b73] Van Buskirk J, Candolin U, Wong BBM (2012). Behavioural plasticity and environmental change. Behavioural responses to a changing world.

[b74] Visser ME, van Noordwijk AJ, Tinbergen JM, Lessells CM (1998). Warmer springs lead to mistimed reproduction in great tits (*Parus major*. Proc. R. Soc. Lond. B.

[b75] Welbergen JA, Davies NB (2008). Reed warblers discriminate cuckoos from sparrowhawks with graded alarm signals that attract mates and neighbours. Anim. Behav.

[b76] Welbergen JA, Davies NB (2009). Strategic variation in mobbing as a front line of defense against brood parasitism. Curr. Biol.

[b77] Welbergen JA, Davies NB (2011). A parasite in wolf’s clothing: hawk mimicry reduces mobbing of cuckoos by hosts. Behav. Ecol.

[b78] Welbergen JA, Davies NB (2012). Direct and indirect assessment of parasitism risk by a cuckoo host. Behav. Ecol.

