# Supplementary material for: Reed Warbler Hosts Fine-Tune their Defenses to Track Three Decades of Cuckoo Decline
Source: Evolution. 2013 Aug 8;67(12):3545–55. doi: 10.1111/evo.12213 (PMC4209118; doi:10.1111/evo.12213)
Supplement: Supplementary file 1 — S1: Data table for Figure 1 S2: Distribution of data for Figure 3 S3: Data table and references for Figures 3 & 4 Table S1. Changes in cuckoo and reed warbler populations (from cuckoo egg observations and standardized nest searching effort), parasitism rate (proportion of all nests found that contained cuckoo eggs), and expression of hosts’ first line of defense (mobbing an adult cuckoo) at Wicken Fen, Cambridgeshire, U.K., from 1985 to 2012. Figure S2. Distribution of parasitism rates during laying in each year we conducted experiments testing (a) mobbing responses and (b) rejection of nonmimetic eggs at reed warbler nests. Table S3. Parasitism rates and proportion of hosts who (a) mob taxidermy adult cuckoo models or (b) reject non-mimetic eggs in other reed warbler populations across Europe. References for Table S3 [file evo0067-3545-sd1.doc]

SUPPORTING INFORMATION FOR:

**REED WARBLER HOSTS FINE-TUNE THEIR DEFENSES TO TRACK THREE DECADES OF CUCKOO DECLINE**

**Rose Thorogood & Nicholas B. Davies**

Department of Zoology, University of Cambridge, Cambridge, U.K.

S1: Data table for Figure 1

S2: Distribution of data for Figure 3

S3: Data table and references for Figures 3 & 4

Table S1: Changes in cuckoo and reed warbler populations (from cuckoo egg observations and standardized nest searching effort), parasitism rate (proportion of all nests found that contained cuckoo eggs), and expression of hosts’ first line of defense (mobbing an adult cuckoo) at Wicken Fen, Cambridgeshire, U.K. from 1985 – 2012. Changes (slope, β, ± S.E.) in parasitism rate and mobbing propensity (number of nests where reed warblers alarm called and/or snapped their bills at model adult cuckoos / number of nests tested, see Methods) were tested with a binomial generalized model; changes in population sizes with a linear regression. Significant changes are shown in bold.

|  | Number of female cuckoos | Index of reed warbler population | Parasitism rate | Proportion of pairs who mob |
| --- | --- | --- | --- | --- |
| 1985 | 14 | 67 | 24.3% (34/140) | 27/28 (96.4%) |
| 1986 | - | 58 | 15.9% (18/113) | 24/25 (96%) |
|  |  |  |  |  |
| 1996 | - | 65 | - | 35/60 (58.3%) |
| 1997 | 7 | 85 | 6.6% (15/225) | 11/14 (78.6%) |
|  |  |  |  |  |
| 2000 | - | - | - | 22/31 (71%) |
| 2001 | 2 | - | 8.4% (9/107) | 11/16 (69%) |
| 2002 | 2 | - | 6.6% (7/106) | 9/12 (75%) |
| 2006 | 2 | 67 | 3.4% (4/118) | 25/48 (52.1%) |
| 2007 | 4 | 54 | 10.4% (11/106) | 11/24 (45.8%) |
| 2008 | 5 | 61 | 15.6% (20/128) | 91/191 (47.6%) |
| 2010 | 1 | 42 | 3.7% (4/107) | 17/36 (47.2%) |
| 2011 | 2 | 66 | 3.1% (5/160) | 16/43 (37.2%) |
| 2012 | 1 | 65 | 1.4% (2/143) | 7/15 (46.7%) |
|  |  |  |  |  |
| β | -0.43 ± 0.08 | -0.29 ± 0.37 | -0.06 ± 0.01 | -0.10 ± 0.02 |
|  | *F* = 25.8 | *F* = 0.64 | χ^2^ = 42.8 | χ^2^ = 55.2 |
| *p* | **0.001** | 0.45 | **<0.0001** | **<0.0001** |

Figure S2: Distribution of parasitism rates during laying in each year we conducted experiments testing (a) mobbing responses and (b) rejection of non-mimetic eggs at reed warbler nests. These data were pooled and used as the x-axis in Figure 3, but using 1985 data only did not change our results (see main text). Heavy line denotes median parasitism rate, boxes show the interquartile range (IQR), whiskers extend to 1.5 x IQR, and outliers are shown as open circles.

(a) mobbing of adult cuckoo models

(b) rejection of non-mimetic eggs

Table S3: Parasitism rates and proportion of hosts who (a) mob taxidermy adult cuckoo models or (b) reject non-mimetic eggs in other reed warbler populations across Europe. Some populations were measured multiple times, but with different experimental methods and in different years (indicated in parentheses). See sources for descriptions of sites and experimental methods and Figure 4 for a display of the locations.

| Location | Parasitism rate (%) | Proportion defends (%) | Source |
| --- | --- | --- | --- |
| *(a)* *mobbing* |  |  |  |
| Palermo, Italy | 27/166 (16) | 72/108 (66.7) | [1,2], D. Campobello |
| Lednice, Czech Republic | 60/399 (15) | 15/42 (35.7) | [3,4], E. Roskaft |
| Llangorse lake, U.K. (2007-09) | 0/136 (0) | 15/60 (25) | [5] |
|  |  |  |  |
| *(b)* *egg rejection* |  |  |  |
| Lužice, Czech Republic (1998) | 11/94 (11.7) | 27/48 (56.3) ^a^ | [6] |
| Lužice, Czech Republic (2002) | 12/82 (14.6) | 13/29 (44.8) ^b^ | [7], B.G. Stokke |
| Arresø, Denmark | 8/99 (8.1) | 18/38 (47.4) ^b^ | [7] |
| Brittany, France | 0/41 (0) | 10/30 (33.3) ^b^ | [7] |
| Bavaria, Germany | 6/72 (8.3) | 31/45 (68.9) ^b^ | [7] |
| Rhineland, Germany | 8/55 (14.5) | 8/24 (33.3) ^b^ | [7] |
| Ventes Ragas, Lithuania | 0/61 (0) | 6/25 (24) ^b^ | [7] |
| Norway | *(0) | 3/26 (11.5) ^a^ | [8] |
| Silesia, Poland | 15/105 (14.3) | 31/52 (59.6) ^b^ | [7] |
| Danube Delta, Romania | 4/69 (5.8) | 23/39 (59) ^b^ | [7] |
| Transylvania, Romania | 0/32 (0) | 9/28 (32.1) ^b^ | [7] |
| Badajoz, Spain | 0/27 (0) | 1/21 (4.8) ^b^ | [7] |
| Ebro Delta, Spain | 4/19 (21.1) | 3/11 (27.3) ^b^ | [7] |
| Madrid, Spain | 0/28 (0) | 3/15 (20) ^b^ | [7] |
| Galicia, Spain | 0/39 (0) | 16/36 (44. 4) ^b^ | [7] |
| Llangorse lake, U.K. (1993) | 0/160 (0) | 5/35 (14.3) ^c^ | [9,10] |
| Llangorse lake, U.K. (2002) | 0/49 (0) | 2/16 (12.5) ^b^ | [7], B.G. Stokke |
| Pannel valley, U.K. | 0/121 (0) | 12/31 (38.7) ^c^ | [9] |
| ^a^ redstart-type model egg; ^b^ redstart-type painted quail’s egg; ^c^ pied wagtail-type model egg  * number of nests not available | | | |

References for Table S3

1 Campobello, D. & Sealy, S. G. 2011 Use of social over personal information enhances nest defense against avian brood parasitism. *Behav. Ecol.* **22**, 422-428.

2 Campobello, D. & Sealy, S. G. 2010 Enemy recognition of reed warblers (Acrocephalus scirpaceus): Threats and reproductive value act independently in nest defence modulation. *Ethology* **116**, 498-508.

3 Røskaft, E., Moksnes, A., Stokke, B. G., Bicik, V. & Moskat, C. 2002 Aggression to dummy cuckoos by potential European cuckoo hosts. *Behaviour* **139**, 613-628.

4 Øien, I., Moksnes, A., Røskaft, E. & Honza, M. 1998 Costs of cuckoo *Cuculus canorus* parasitism to reed warblers *Acrocephalus scirpaceus*. *J. Avian Biol.* **29**, 209–215.

5 Welbergen, J. A. & Davies, N. B. 2012 Direct and indirect assessment of parasitism risk by a cuckoo host. *Behav. Ecol.* **23**, 783-789.

6 Stokke, B. G., Moksnes, A., Røskaft, E., Rudolfsen, S. & Honza, M. 1999 Rejection of artificial cuckoo (*Cuculus canorus*) eggs in relation to variation in egg appearance among reed warblers (*Acrocephalus scirpaceus*). *Proc. R. Soc. B* **266**, 1483–1488.

7 Stokke, B. G., Hafstad, I., Rudolfsen, G., Moksnes, A., Møller, A. P., Røskaft, E. & Soler, M. 2008 Predictors of resistance to brood parasitism within and among reed warbler populations. *Behav. Ecol.* **19**, 612-620.

8 Avilés, J. M., Stokke, B. G., Moksnes, A., Røskaft, E., Asmul, M. & Møller, A. P. 2006 Rapid increase in cuckoo egg matching in a recently parasitized reed warbler population. *J. Evolution. Biol.* **19**, 1901-10.

9 Lindholm, A. K. & Thomas, R. J. 2000 Differences between populations of reed warblers in defences against brood parasitism. *Behaviour* **137**, 25-42.

10 Lindholm, A. K. 1999 Brood parasitism by the cuckoo on patchy reed warbler populations in Britain. *J. Anim. Ecol.* **68**, 293-309.
